# A half doubling dose change in bronchial hyperresponsiveness in a population represents an important difference

**DOI:** 10.1186/2213-0802-1-4

**Published:** 2013-02-27

**Authors:** Mark Weatherall, James Fingleton, Sally Eyers, Richard Beasley

**Affiliations:** 1 University of Otago Wellington, Wellington, New Zealand; 2 Medical Research Institute of New Zealand, Private Bag 7902, Newtown, Wellington, 6242 New Zealand

**Keywords:** Asthma, Bronchial hyperresponsiveness, Bronchial hyperreactivity, Medication use, Outcome assessment (Health care)

## Abstract

**Background:**

The prevalence of asthma has increased over recent decades and the reasons for this are poorly understood. A sensitive tool that can evaluate potential risk factors for asthma is bronchial hyperresponsiveness (BHR), a key physiological characteristic of asthma. However, although the minimum clinically important difference in BHR for an individual is accepted to be around one doubling dose, the minimum important change in a population is not defined. As with surrogate measures of cardiovascular disease risk such as blood pressure and cholesterol, a change that is not clinically important in an individual may be extremely important in public health terms.

**Findings:**

To assess the potential impact of a small absolute change in BHR across a population, we modelled the effect of different changes in BHR on the prevalence rates of moderate and severe BHR in an asthmatic population. We calculate that a one half doubling dose increase in BHR increases the prevalence of moderate and severe BHR by 30%. If this was accompanied by an equivalent increase in the population prevalence of moderate and severe asthma, this would be highly significant in public health terms.

**Conclusions:**

We propose that a one half doubling dose worsening in BHR across a population may represent an important change.

**Electronic supplementary material:**

The online version of this article (doi:10.1186/2213-0802-1-4) contains supplementary material, which is available to authorized users.

## Findings

### Introduction

The prevalence of asthma and severe asthma has increased over recent decades and the reasons for this are poorly understood [1–3]. Large observational studies such as the ISAAC programme highlight many potential contributions to the increased rate of asthma including changes in microbial [4] and parasitic exposure in childhood [5], dietary habits [6] and environmental conditions[4, 5], as well as use of medications such as paracetamol [7–9]. Observational studies cannot prove causality and randomised controlled trials are better to evaluate the potential role of risk factors in the development of asthma and its severity. A sensitive tool to assess the effect of potential risk factors is bronchial hyperresponsiveness (BHR), a key underlying physiological characteristic of asthma [10]. Despite the common use of BHR testing in clinical trials, the minimum important difference for a population is not known.

**Figure 1:**
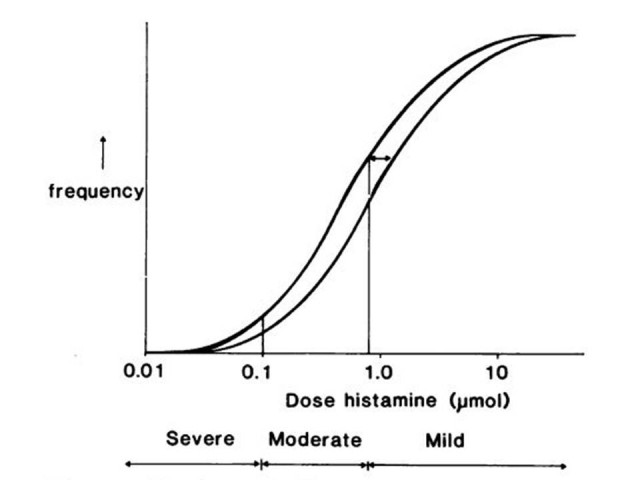
**Frequency distribution of asthma severity as measured by the cumulative dose of histamine which causes a 20% fall in FEV**_**1**_ **.** The small change in bronchial hyperresponsiveness (↔) in the population results in a small increase in the proportion of patients with moderate asthma but a substantial increase in the proportion with severe asthma. [Reproduced from Reference [12].

BHR can be assessed through direct or indirect challenge testing. Although indirect testing may be more closely related to the degree of airway inflammation, BHR is most commonly measured through a direct bronchial challenge test using histamine or methacholine. Direct bronchial challenge testing is the preferred way of assessing the effect of medications in clinical trials [11]. With direct BHR the patient is exposed to progressively greater concentrations of histamine or methacholine, with each step double the dose or concentration of the previous one. BHR is then usually expressed in terms of the dose or concentration required to reduce the forced expiratory volume in one second (FEV1) by 20% (PD_20_ or PC_20_). A relatively small change in BHR which may be of little significance for the individual patient, and even undetectable in view of the natural variability and severity seen in asthma, may, when applied to the whole population of patients with asthma, represent a change of major significance [Figure 1] [12]. This is analogous to other surrogate markers of cardiovascular disease risk such as blood pressure or cholesterol; in which it has been clearly demonstrated that small changes, which are unlikely to be of significance to an individual and are smaller than between test variability, can change the incidence of disease when replicated across a population. For example it has been suggested that a 2 mmHg reduction in diastolic blood pressure across the United States population, although unimportant at an individual level, would lead to a 6% reduction in coronary heart disease and 15% reduction in stroke [13].

To assess the potential impact of a small absolute change in BHR in a population with asthma, we modelled the effect of different changes of BHR on the prevalence rates of moderate and severe BHR.

## Methods

BHR prevalence data was extracted from the report of a population study by Woolcock and colleagues [14] who studied the population distribution of BHR, measured as PD_20_ to histamine, in a random sample of a rural adult population. Data was presented on the frequency of BHR, below a cut-off value for whether BHR was present or not. The PD_20_ was calculated as the cumulative dose of histamine causing a 20% fall in FEV1 with ‘cut-off’ values of 0.1 μmol and 1.0 μmol histamine defining severe and moderate BHR respectively [14].

Extracted data were plotted as cumulative frequency versus histamine dose to assess fit with the expected sigmoid function and then histamine dose was expressed as logarithm base two, so equal distances between histamine doses represent doubling doses, and the cumulative prevalence was expressed on the natural logarithm scale. The transformed variables plot was linear and regression was used to estimate the change in cumulative prevalence of BHR prevalence rates in relation to a doubling dose change of PD_20_ (histamine).

## Results

The plot of cumulative frequency versus histamine dose closely resembled the left hand region of a sigmoid curve, as in the figure. The slope of the line of the transformed variables relating logarithm cumulative prevalence of BHR to a doubling dose change of histamine PD_20_ is 0.55 (95% CI 0.47 to 0.62). By exponentiation this represents a change in cumulative prevalence rate ratio of 1.73 (95% CI 1.60 to 1.86) per doubling dose change in BHR.

BHR to histamine was present in 10.5% of the population in the Woolcock study[14]. Within this group severe BHR (defined as a PD_20_ (histamine) ≤0.1 μmol), was present in 6.6%, and moderately severe BHR (defined as a PD_20_ (histamine) of up to 1.0μmol), was present in 41.2%. Based on these figures, the changes in prevalence of severe and moderately severe BHR expected from different doubling dose changes in population BHR are estimated and shown in Table 1. A one half doubling dose worsening in BHR increases the prevalence of severe BHR from 6.6% to 8.6% (95% CI 8.3%-9.0%) and moderate to severe BHR from 41.2% to 54.0% (95% CI 51.9%-56.0%), a relative increase of approximately 30%.

**Table 1:** **Predicted prevalence of severe and moderately severe BHR with changes in population BHR [Based on data from reference** [14]**]**

Doubling dose worsening in population BHR	Change in Prevalence Rate ratio (95% CI)	Prevalence of moderately severe BHR, % (95% CI)	Prevalence of severe BHR, % (95% CI)
Baseline	-	41.2	6.6
0.25	1.15 (1.12-1.17)	47.3 (46.3-48.1)	7.6 (7.4-7.7)
0.5	1.31 (1.26-1.36)	54.2 (52.1-56.2)	8.7 (8.3-9.0)
0.75	1.51 (1.42-1.59)	62.2 (58.6-65.6)	10.0 (9.4-10.5)
1.0	1.73 (1.60-1.86)	71.4 (65.9-76.6)	11.4 (10.6-12.3)

## Discussion

Whilst a precise definition of minimum important difference in BHR for a population is inherently somewhat arbitrary, our findings suggest that a one half doubling dose increase in BHR could significantly alter the prevalence of severe BHR within a population. Our calculations indicate that a half doubling dose increase in BHR leads to a 30% relative increase in the prevalence of severe and moderately severe BHR, with absolute increases of 2% and 13% respectively. In contrast a quarter doubling dose worsening in BHR leads to only a minor change, with the 95% confidence interval encompassing a change in the prevalence of severe BHR of <1%.

A limitation of the interpretation of this analysis is that an increase in the prevalence of severe BHR cannot be assumed to lead to an equivalent increase in the prevalence of severe asthma. However, increased sensitivity to bronchial stimuli is one of the fundamental mechanisms of asthma, [11] increased BHR is associated with more severe asthma [15] and BHR is a recognised outcome measure reflecting disease activity in asthma [11, 16].

If the increases in BHR modelled here result in a similar increase in the prevalence of moderate to severe asthma this would be important in public health terms. In the same way that risk factors such as salt can cause small changes in blood pressure across a population, it is possible that small individual changes in BHR caused by environmental exposure or medication use may lead to substantial differences in asthma severity across a population. We therefore propose that a half doubling dose worsening in population BHR in response to a risk factor such as medication use represents an important and meaningful change.

## Authors’ original submitted files for images

Below are the links to the authors’ original submitted files for images. Authors’ original file for figure 1

## Abbreviations

BHR: Bronchial Hyperresponsiveness
FEV1: Forced Expiratory Volume in 1 second
ISAAC: International Study of Asthma and Allergies in Childhood
PD20/PC20: The dose or concentration required to reduce the forced expiratory volume in one second (FEV1) by 20%
