# Supplementary figures and images for: A half doubling dose change in bronchial hyperresponsiveness in a population represents an important difference
Source: Transl Respir Med. 2013 Feb 27;1:4. doi: 10.1186/2213-0802-1-4 (PMC6733426; doi:10.1186/2213-0802-1-4)

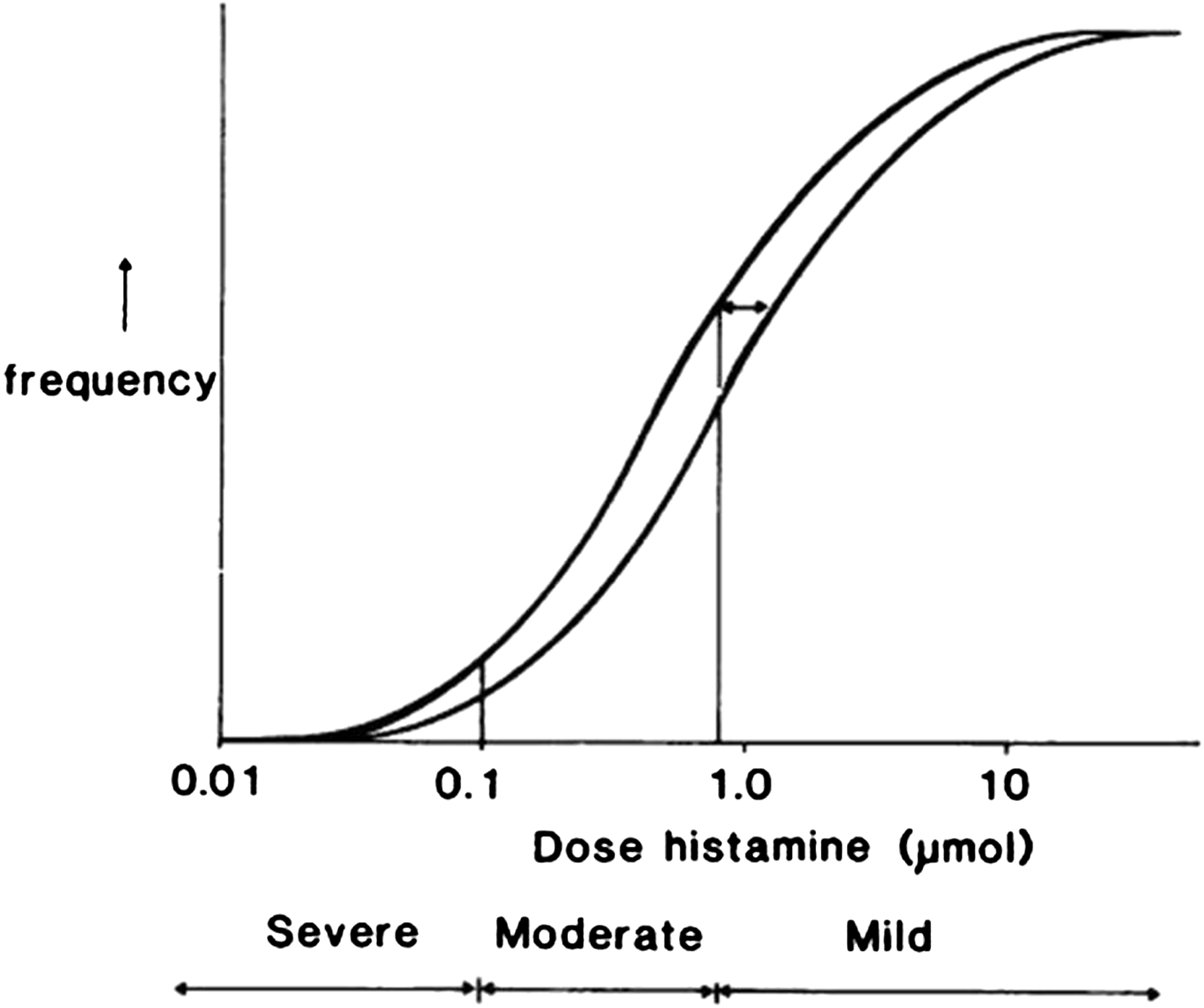

Supplement: Supplementary file 1 — Authors’ original file for figure 1 [file 40247_2012_4_MOESM1_ESM.tiff]
